# Triple Combination Therapy With PD-1/PD-L1, BRAF, and MEK Inhibitor for Stage III–IV Melanoma: A Systematic Review and Meta-Analysis

**DOI:** 10.3389/fonc.2021.693655

**Published:** 2021-06-14

**Authors:** Ye Liu, Xilan Zhang, Guoying Wang, Xinchang Cui

**Affiliations:** ^1^ School of Pharmaceutical Sciences, Tsinghua University, Beijing, China; ^2^ Department of Pathology, The Second People’s Hospital of Dongying, Dongying, China; ^3^ Department of Oncology, The Second People’s Hospital of Dongying, Dongying, China

**Keywords:** triple combination therapy, PD-1/PD-L1, BRAF inhibition, MEK inhibition, melanoma, meta-analysis

## Abstract

Triple combination of anti-PD-1/PD-L1 immunotherapy and anti-BRAF plus anti-MEK targeted therapy is a promising antitumor strategy and is increasingly being used in clinical trials. To evaluate the safety and efficacy of triple combination of PD-1/PD-L1, BRAF, and MEK inhibition in patients diagnosed with stage III-IV melanoma, we performed a systematic review and meta-analysis of randomized controlled trials (RCTs). The PubMed, EMBASE, and Cochrane Library were searched for all studies published from inception to January 2021. The progression free survival (PFS), overall survival (OS), overall response rate (ORR), and risk of adverse events (AEs) were extracted by two independent investigators and pooled hazard ratio (HR) or risk ratio (RR) with 95% CI were determined using the random-effects model for data synthesis. Overall, five randomized controlled trials encompassing 1,266 patients with stage III-IV melanoma were selected. Triple combination therapy significantly improved PFS (HR = 0.71; 95% CI = 0.59 to 0.86; P = 0.0005) and 2-year OS (RR = 1.12; 95% CI = 1.03 to 1.23; P = 0.01), but had no impact on ORR (RR = 1.09; 95% CI = 0.91 to 1.30; P = 0.37) when compared with controlled treatment group. In addition, triple combination therapy was associated with increased risks of hypothyroidism, arthralgia, myalgia, ALT increased, AST increased, asthenia, and pyrexia compared with control group. Triple combination therapy of PD-1/PD-L1, BRAF, and MEK inhibition achieved better survival benefits but had higher incidence of some adverse events over two-drug combination or monotherapy. Further randomized controlled clinical trials are needed to verify our results.

**Systematic Review Registration:**

PROSPERO 2021 CRD42021235845 Available from https://www.crd.york.ac.uk/prospero/display_record.php?ID=CRD42021235845.

## Introduction

Melanoma is a very aggressive form of skin cancer with the fastest growing incidence rate among all cancers. About 1.7% of all cases of newly diagnosed malignant cancers are cutaneous melanoma (1, 2). Once melanoma has spread, it becomes the deadliest type of cutaneous cancer. As a result of its high heterogeneity and ability to the elude the body’s immune system, melanoma cells often display a multidrug resistance phenotype and are extremely difficult to treat (3, 4).

Recently, the therapies for patients with advanced melanoma have been upgraded with the development of small molecule inhibitors targeting B-Raf proto-oncogene serine/threonine-kinase (BRAF) and/or MAPK/ERK kinase (MEK), as well as immunotherapy drugs targeting the programmed death-1/programmed death-ligand-1 (PD-1/PD-L1) and the cytotoxic T lymphocyte antigen-4 (CTLA-4) (5, 6). Targeted therapy including a BRAF inhibitor (vemurafenib, dabrafenib, and encorafenib) in combination with a MEK inhibitor (trametinib, cobimetinib, binimetinib, and selumetinib) was the first-line therapy for metastatic or advanced melanoma (7, 8). With deeper understanding of cancer immunology and immunotherapy, PD-1/PD-L1 immune checkpoint inhibitors (pembrolizumab, pidilizumab, nivolumab, atezolizumab, durvalumab, avelumab, or spartalizumab), and CTLA-4 inhibitors also served as first-line therapy for advanced melanoma (2, 9, 10).

Immunotherapy and targeted therapy both have significant advantages and disadvantages. A benefit of targeted therapy over immunotherapy is the high objective response rates, which means more people respond to targeted therapy. However, the main disadvantage is that the duration of response (DOR) is short-lived (11). A clear advantage of immunotherapy over targeted therapy is providing more durable responses and inhibitory effects on cancer growth which may continue to exist after the drugs have been discontinued (12). However, the biggest drawback is the relatively lower response rate, as only a small percentage of people respond to immunotherapy (13). Thus, there is a continuous need to develop new treatment strategies.

Considering that immunotherapy and targeted therapy are complementary in terms of advantages and disadvantages, combinations of immunotherapy and targeted therapy are proposed and applied to clinical trials. Data show that treatment with BRAF and MEK inhibitors increases T cell numbers, CD8^+^ T cell infiltration, downregulates immunosuppressive cytokines and upregulates PD-1/PD-L1 expressions (14, 15), implying that adding PD-1/PD-L1 inhibitor to BRAF and MEK targeted therapy could help to prevent the spread of cancer. Despite some exciting results from the recent clinical studies (16–18), the question of whether triple combination therapy is better than other drug therapy is still open. To solve this puzzle, we conducted a systematic review and meta-analysis of clinical trials to determine whether the triple therapy combined with PD-1/PD-L1, BRAF, and MEK inhibitor has better outcomes than two-drug combination or monotherapy in metastatic melanoma treatment.

## Methods

The study was performed in accordance with the PRISMA (Preferred Reporting Items for Systematic Reviews and Meta-analyses) guidelines (19). The protocol was registered in PROSPERO (CRD42021235845).

### Search Strategy

Literature searches were done without language restrictions using PubMed, EMBASE, and Cochrane Library from inception to January 2021. The key search terms with Boolean operators (AND, OR) were as follows: (“melanoma” OR “skin neoplasms”) AND (“PD-1” OR “PD-L1” OR “pembrolizumab” OR “pidilizumab” OR “nivolumab” OR “atezolizumab” OR “durvalumab” OR “avelumab” OR “spartalizumab”) AND (“BRAF” OR “vemurafenib” OR “zelboraf” OR “dabrafenib” OR “encorafenib”) AND (“MEK” OR “trametinib” OR “cobimetinib” OR “binimetinib” OR “selumetinib”). The detailed search strategies for each database are available in **Table S1**.

### Selection Criteria

Titles and abstracts were initially screened for relevance, and then full-text screening was carried out. Screening was performed in duplicate by two investigators (YL and XLZ). Studies were considered eligible if the selection criteria were met: (1) The study design of literature was a randomized clinical trial. (2) Enrolled patients with stage III-IV melanoma had histologically confirmed diagnosis of unresectable Stage III or metastatic stage IV melanoma and had at least one measurable lesion as defined by RECIST 1.1 on CT or MRI imaging studies. (3) Intervention treatments were triple combination therapy combined with PD-1/PD-L1 inhibition, BRAF inhibition and MEK inhibition versus two-drug combination therapy or monotherapy. (4) Study results included progression-free survival (PFS), overall survival (OS), overall response rate (ORR), and adverse events (AEs). Studies with unavailable or incomplete data were excluded.

### Data Extraction

Two investigators (YL and GW) independently reviewed the full text of eligible studies and extracted data using a prespecified data-collection form, including the following information: publication reference, study type, clinical trial registry numbers, sample size, age, gender, treatment regimen, and outcomes (including PFS, OS, ORR and AEs). For the IMPemBra study [Rozeman et al. (20)] and COMBI-i study [Nathan et al. (21)], there were only descriptive words of outcomes without detailed survival curves and adverse events, therefore, we referred to the clinical data presented at American Society of Clinical Oncology (ASCO) and European Society for Medical Oncology (ESMO) conferences, respectively. When data of PFS and 2-year OS were not reported in the text, it was independently calculated from survival curves using graph data extraction software Engauge Digitizer.

### Quality Assessment

Methodologic quality of included studies was assessed independently by two investigators (GYW and XCC) using the Cochrane Collaboration’s risk-of-bias tool (22). Bias assessment was generated by Review Manager Version 5.4 (Cochrane Collaboration).

### Data Analysis

The risk ratio (RR) with 95% confidence interval (CI) was used to summarize the dichotomous outcomes, including 2-year OS, ORR, complete response (CR), partial response (PR), and adverse events (i.e., nausea, arthralgia, diarrhea, asthenia), while hazard ratio (HR) with 95% CI was used to summarize results for PFS. We generated forest plots using Review Manager 5.4. The random-effects model was used for all meta-analysis, and a value of *P* < 0.05 was considered as statistically significant. Heterogeneities between the studies were evaluated by I^2^ statistic, and I^2^ > 50% represented significant heterogeneity (23). Sensitivity analysis was conducted using Stata/SE 16.0 to evaluate the influence of every single study on the overall estimate *via* omitting study in turn. Because of the limited number (<10) of included studies, we did not assess the publication bias.

## Results

### Included Trials and Studies

**Figure 1:**
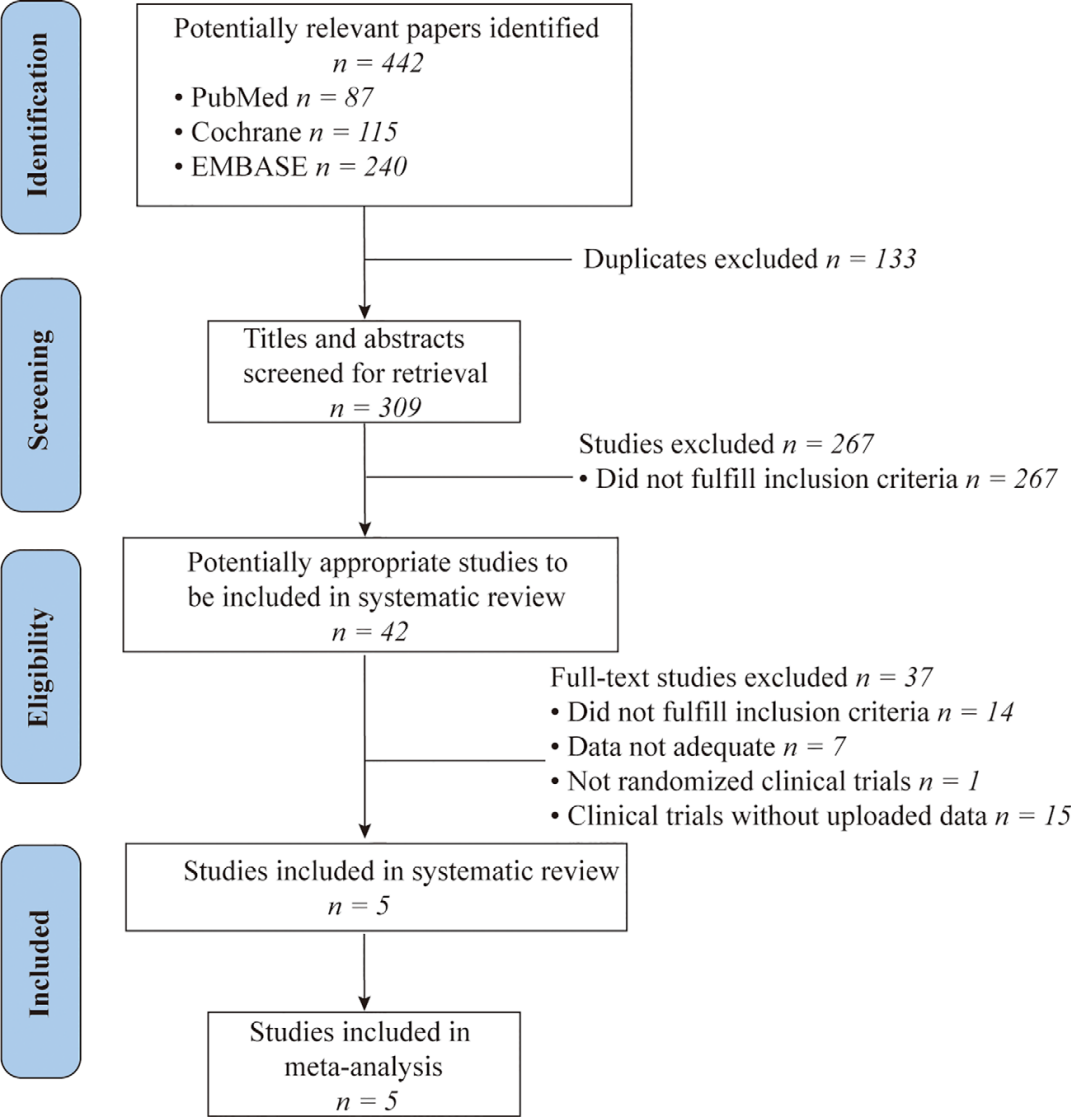
A PRISMA Flow chart of study selection. PubMed, EMBASE and Cochrane Library databases were searched for articles published from inception to January 1, 2021.

A total of 442 relevant citations were initially retrieved from PubMed, EMBASE, and Cochrane Library databases. After duplicate checking, 309 studies were included. Then, 267 studies were excluded based upon the assessment of titles or abstracts and because of them being reviews, comments, case reports, animal trials, or irrelevant to our inclusion criteria. Screening of the full-text citations resulted in the exclusion of studies that did not fulfill the inclusion criteria (n=14); did not show adequate data (n=7); not randomized clinical studies (n=1) or in the early stage of clinical trials without uploaded results (n=15). Finally, five randomized clinical trials (RCTs) were included in our meta-analysis (20, 21, 24–26)and details about selection of studies were schematically shown in **Figure 1**.

### Study Characteristics

All the included randomized trials were published in 2020. Of all eligible studies, we gathered a total of 1,266 patients with stage III to IV metastatic melanoma treated with triple therapy of PD-1/PD-L1 inhibition, BRAF inhibition, and MEK inhibition *versus* two-drug combination or monotherapy as control group. The detailed characteristics of these trials were presented in **Table 1**. In terms of patient characteristics, all studies except one enrolled *BRAF* ^V600^ mutation-positive patients in both triple and control treatment group. Only a study by Ribas et al. enrolled *BRAF* ^V600^ mutation-positive patients in triple therapy group, while *BRAF*-wild type patients and a patient with other mutation in two-drug control group. In terms of treatment regimen, patients of the IMSpire 150 study in triple therapy arms were treated with atezolizumab (PD-L1 inhibitor) in combination with vemurafenib and cobimetinib; Ribas et al. study used durvalumab (PD-L1 inhibitor) combined with dabrafenib and trametinib; patients of the Keynote-022 and IMPemBra study received pembrolizumab (PD-1 inhibitor) in combination with dabrafenib and trametinib; and COMBI-i study used PD-1 inhibitor spartalizumab in combination with dabrafenib and trametinib. Besides that, only a study by Rozeman et al. used pembrolizumab monotherapy as control group, while IMSpire 150, Keynote-022, and COMBI-i studies all used BRAF inhibitor plus MEK inhibitor as controlled two-drug combination treatment and Ribas et al. study applied PD-L1 inhibitor and MEK inhibitor in control arm.

**Table 1:** The main characteristics of 5 included studies in the meta-analysis.

Study	Trial	Study type	Melanoma severity	Triple group Control	Sample size	Age (year)	Female, n%	Treatment regimen
Gutzmer et al. (25)	NCT02908672, IMSpire 150	Phase III RCT	Stage IIIc-IV, BRAF^V600^ mutation-positive	ate+vem+cob	256	54.0 (44.8-64.0)	106, 41%	Vem 720mg BID + Cob 60mg QD + Ate 840mg
				vem+cob	258	53.5 (43.0-63.8)	109, 42%	Vem 960mg BID + Cob 60mg QD + placebo
Ferrucci et al. (24)	NCT02130466, KEYNOTE-022	Phase II RCT	Stage III-IV, BRAF^V600^ mutation-positive	pem+dab+tra	60	54 (18-82)	27, 45.0%	Pem Q3W+ Dab 150mg BID + Tra 2mg QD
				dab+tra	60	58 (21-83)	24, 40.0%	Placebo Q3W+ Dab 150mg BID + Tra 2mg QD
Rozeman et al. (20)	NCT02625337, IMPemBra	Phase II RCT	Stage IIIc-IV, BRAF^V600^ mutation-positive	pem+dab+tra	24	56 (22-78)	12, 50%	Pem 200mg Q3W + Dab 150mg BID + Tra 2mg QD
				pem	8	58 (46-71)	2, 25%	Pem 200mg Q3W
Nathan et al. (21)	NCT02967692, COMBI-i	Phase III RCT	Stage IIIc-IV, BRAF^V600^ mutation-positive	spa+dab+tra	267	56 (20-86)	225, 42.3%	Spa 400mg Q4W + Dab 150mg BID + Tra 2mg QD
				dab+tra	265	55 (23-88)		Placebo Q4W + Dab 150mg BID + Tra 2mg QD
Ribas et al. (26)	NCT02027961	Phase I RCT	Stage III-IV melanoma	dur+dab+tra	26	49.0 (23-71)	12, 46.2%	Dur 3 or 10mg/kg Q2W + Dab 150mg BID + Tra 2mg QD
				dur+tra	42	n=20: 68.0 (31-85) n=22: 63.0 (34-84)	18, 42.9%	Dur 10mg/kg Q2W + Tra 2mg QD (Concurrent n=20; Sequential n=22)

Data of age were presented as median (range). ate, atezolizumab; vem, vemurafenib; cob, cobimetinib; pem, pembrolizumab; dab, dabrafenib; tra, trametinib; spa, spartalizumab; dur, durvalumab. BID, twice daily; QD, once daily; Q2W, every 2 weeks; Q3W, every 3 weeks; Q4W, every 4 weeks.

### Risk-of-Bias Assessment

**Figure 2:**
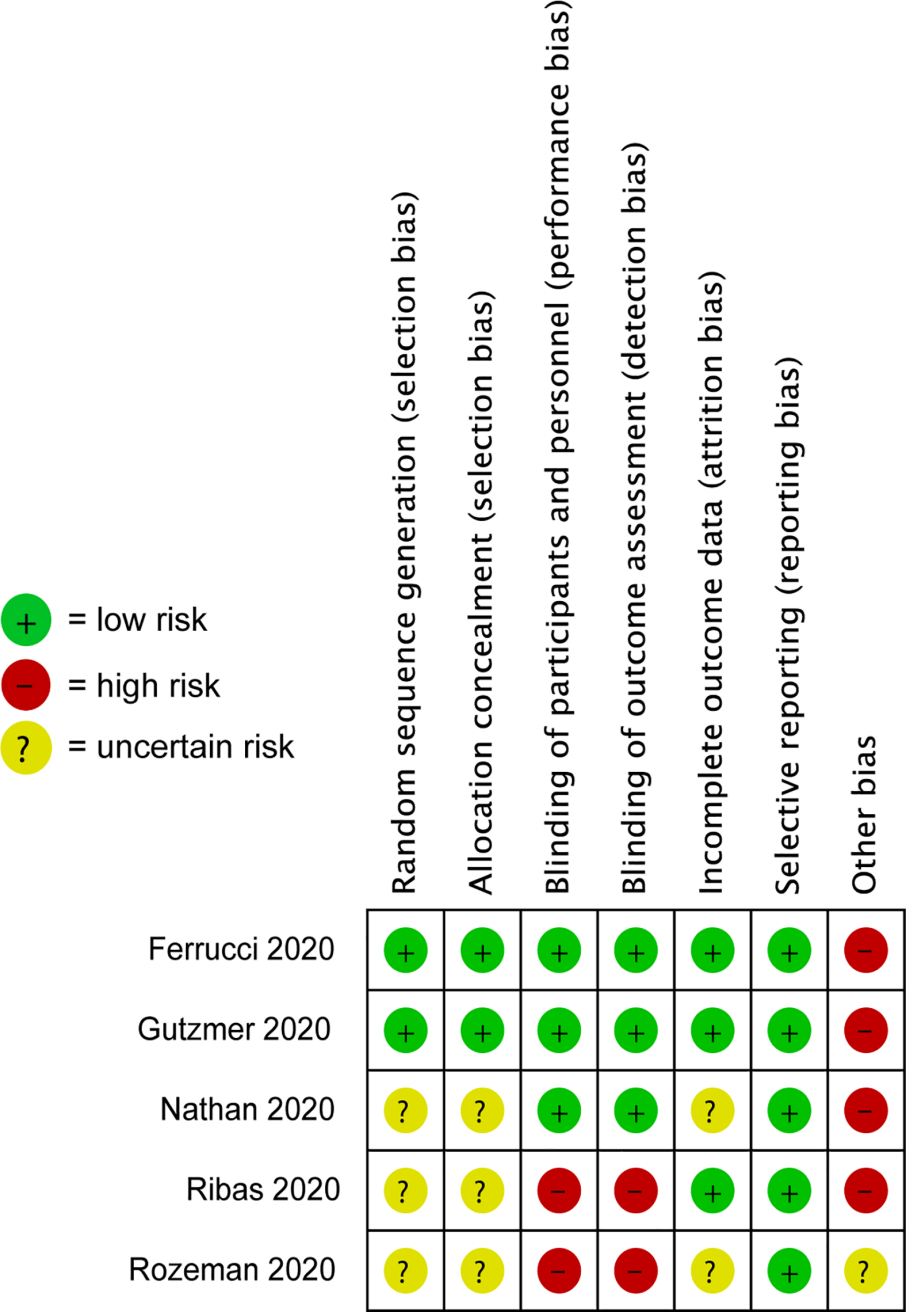
Risk-of-bias assessment of randomized controlled trials (RCTs) included in meta-analysis.

The risk of bias assessment of the included studies has been listed in **Figure 2**. Three trials, including Ferrucci et al., Gutzmer et al., and Nathan et al., were double-blind studies, whereas the study by Ribas et al. and Rozeman et al. were open‐label. Four studies were at high risk of other bias, because pharmaceutical companies either sponsored these clinical trials or provided the study drugs.

### Progression-Free Survival

**Figure 3:**
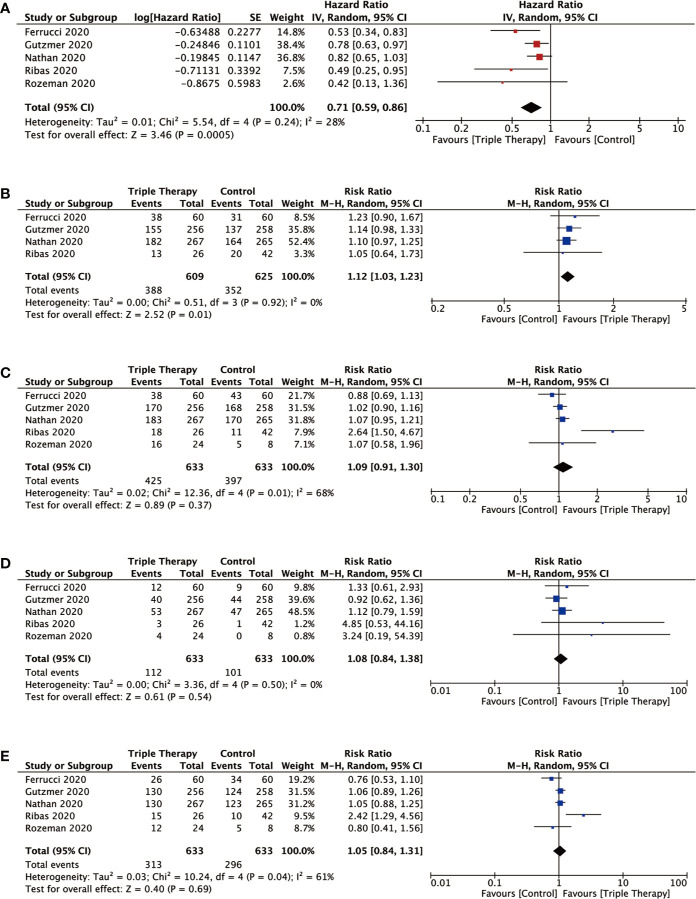
Forest plots analysis of the efficiency outcomes for triple combination therapy of PD-1/PD-L1, BRAF, and MEK inhibition versus control therapy. Pooled hazard ratio (HR) or risk ratio (RR) with 95% CI were determined using the random-effects model for outcomes. **(A)** PFS (progression-free survival); **(B)** OS (overall survival); **(C)** ORR (overall response rate); **(D)** CR (complete response); **(E)** PR (partial response).

Forest plot of PFS related to triple therapy and control therapy was shown in **Figure 3A**. The information about HRs for PFS was available from trials or calculated from survival curves given in studies. The pooled HR for PFS based on our random-effects model analysis indicated that triple combination therapy was associated with significantly longer PFS as compared to control (HR = 0.71; 95% CI = 0.59 to 0.86; *P* = 0.0005; I^2^ = 28%).

### Overall Survival

The risk ratio (RR) for 2-year OS was 1.12 (95% CI = 1.03 to 1.23; *P* = 0.01; I^2^ = 0%). For the triple therapy group, the pooled 2-year OS rate estimate was 63.7% (n=388/609 responses) and for the control group, it was 56.3% (n=352/625 responses). *P* value of 2-year OS outcomes demonstrated statistical difference between triple therapy and control therapy (**Figure 3B**).

### Objective Response Rate, Complete Response, and Partial Response

Forest plots of ORR, CR, and PR associated with triple therapy and control therapy were showed in **Figures 3C–E**. The overall ORR was 67.1% (n=425/633 responses; CR 112; PR 313) in triple therapy and 62.7% (n=397/633 responses; CR 101; PR 296) in control group. However, triple therapy had no significant improvement compared with control and the risk ratio (RR) for ORR was 1.09 (95% CI = 0.91 to 1.30; *P* = 0.37; I^2^ = 68%). In addition, there was no clear benefit for triple therapy in CR (RR = 1.08; 95% CI = 0.84 to 1.38; *P*= 0.54; I^2^ = 0%) and PR (RR = 1.05; 95% CI = 0.84 to 1.31; *P*= 0.69; I^2^ = 61%).

### Adverse Events

Despite the improved survival benefit associated with triple therapy, concerns of AEs, especially the immune-related adverse events (irAEs), occurring during triple combination treatments are growing because of their functional mechanisms (27). In this study, we analyzed the immune-related adverse events and other AEs for all grades. We classified a total of 18 different types of adverse events that were mentioned in the included studies and the statistical details were listed in **Table 2**. There was no significant difference in the incidence of adverse events for all grades (any events, RR = 1.01; 95% CI = 0.99 to 1.04; *P*= 0.39; I^2^ = 47%) and grade≥3 (RR = 1.21; 95% CI = 0.99 to 1.49; *P*= 0.07; I^2^ = 74%) between the triple therapy and control group. For dermatologic irAEs, similar incidence of rash, pruritus, and dermatitis acne occurred in both triple therapy and control group. For gastrointestinal irAEs, triple therapy also showed similar risk of nausea, diarrhea, and vomiting in both triple and control groups. For endocrine irAEs, triple therapy had the higher risk of hypothyroidism than control. For musculoskeletal AEs, triple therapy was associated with more frequent incidences of arthralgia and myalgia between triple therapy and control. Analysis results of hepatic irAEs, such as ALT increased and AST increased in triple therapy showed significant toxicity incidences than control, while blood ALP increased showed no significant toxicity event difference. For general disorders, no significant toxicity event difference was shown in chills, fatigue, headache, and decreased appetite between triple therapy and control group. Moreover, the number of patients experiencing asthenia and pyrexia were greater in triple therapy. In conclusion, our random-effects model analysis revealed that triple therapy could significantly increase the incidence of hypothyroidism, arthralgia, myalgia, ALT increased, AST increased, asthenia and pyrexia (*P* < 0.05).

**Table 2:** Outcomes of all-grade adverse events (AEs) and grade ≥ 3 adverse events for triple combination therapy versus control therapy.

Adverse events	No. studies	RR, 95%CI	P value	Heterogeneity	
				I^2^	P value
Any events	4	1.01 [0.99, 1.04]	0.39	47%	0.13
Grade ≥3	5	1.21 [0.99, 1.49]	0.07	74%	0.004
Rash	5	1.04 [0.89, 1.22]	0.58	0%	0.72
Dermatitis acne	3	1.04 [0.71, 1.52]	0.84	5%	0.35
Pruritus	4	1.20 [0.83, 1.75]	0.33	8%	0.35
Nausea	5	1.14 [0.82, 1.58]	0.43	52%	0.08
Diarrhea	5	1.20 [0.86, 1.69]	0.28	66%	0.02
Vomiting	4	1.20 [0.75, 1.93]	0.45	35%	0.2
Hypothyroidism	3	2.74 [1.64, 4.56]	0.0001	0%	0.76
Arthralgia	5	1.57 [1.04, 2.37]	0.03	67%	0.02
Myalgia	3	1.57 [1.10, 2.24]	0.01	0%	0.63
ALT increased	4	1.54 [1.12, 2.13]	0.009	8%	0.35
AST increased	4	1.43 [1.03, 1.98]	0.03	7%	0.36
Blood ALP increased	2	0.98 [0.67, 1.44]	0.92	0%	0.43
Chills	3	1.74 [0.81, 3.75]	0.15	85%	0.001
Fatigue	5	1.13 [0.84, 1.51]	0.42	57%	0.05
Asthenia	4	1.32 [1.05, 1.67]	0.02	0%	0.63
Pyrexia	3	1.86 [1.17, 2.95]	0.009	86%	0.001
Headache	3	2.16 [0.69, 6.76]	0.18	63%	0.07
Decreased appetite	2	0.95 [0.61, 1.47]	0.82	0%	0.39

ALP, alkaline phosphatase; ALT, alanine aminotransferase; AST, aspartate aminotransferase.

### Sensitivity Analysis

To ensure the robustness of the findings, we further conducted the sensitivity analysis of outcomes to evaluate the influence of every single study on overall results and detect the source of heterogeneity. The sensitivity analyses revealed stable results for PFS, ORR, CR, and PR, with results relatively consistent to those of the pooled outcomes in all included studies (**Table 3** and **Figures S1** **–** **S7**). However, omitting Gutzmer et al. study showed impact on the 2-year OS rate (*P* value for significance changed from 0.01 to 0.06) and AE-all grade (*P* value changed from 0.39 to 0.04), which indicated that this study might influence the pooled results of these two outcomes mentioned above. When omitting Ribas et al. study in AE-grade≥3 results, *P* value decreased from 0.07 to 0.03 with significance.

**Table 3:** Sensitivity analysis of efficiency outcomes (PFS, OS, ORR, CR, PR) and adverse events (AEs-all grade, AEs-grade ≥ 3).

	Outcomes, heterogeneity						
	PFS, I^2^	OS, I^2^	ORR, I^2^	CR, I^2^	PR, I^2^	AE-all grade, I^2^	AE-grade≥3, I^2^
**All studies**	0.71 [0.59, 0.86], 28%	1.12 [1.03, 1.23], 0%	1.09 [0.91, 1.30], 68%	1.08 [0.84, 1.38], 0%	1.05 [0.84, 1.31], 61%	1.01 [0.99, 1.04], 47%	1.21 [0.99, 1.49], 74%
**Study omitted**							
Ferrucci et al. (24)	0.77 [0.66, 0.90], 3%	1.11 [1.01, 1.23], 0%	1.16 [0.93, 1.43], 71%	1.06 [0.81, 1.38], 2%	1.12 [0.89, 1.42], 58%	1.01 [0.99, 1.04], 64%	1.12 [0.98, 1.27], 48%
Gutzmer et al. (25)	0.63 [0.46, 0.88], 42%	1.11 [1.00, 1.25], 0%	1.17 [0.86, 1.61], 75%	1.20 [0.88, 1.65], 0%	1.07 [0.73, 1.56], 71%	1.02 [1.00, 1.05], 0%	1.35 [0.93, 1.96], 75%
Nathan et al. (21)	0.64 [0.48, 0.84], 30%	1.15 [1.01, 1.31], 0%	1.16 [0.84, 1.58], 76%	1.09 [0.72, 1.64], 9%	1.07 [0.73, 1.56], 71%	1.00 [0.98, 1.01], 20%	1.28 [0.88, 1.86], 78%
Ribas et al. (26)	0.74 [0.62, 0.89], 25%	1.13 [1.03, 1.23], 0%	1.03 [0.95, 1.11], 0%	1.06 [0.83, 1.36], 0%	1.01 [0.89, 1.14], 4%	–	1.30 [1.04, 1.62], 77%
Rozeman et al. (20)	0.72 [0.59, 0.88], 35%	–	1.09 [0.90, 1.33], 76%	1.07 [0.84, 1.37], 0%	1.08 [0.84, 1.37], 69%	1.01 [0.99, 1.03], 46%	1.19 [0.98, 1.45], 78%

## Discussion

This meta-analysis combines all currently available data from previous trials to compare a triple combination of BRAF inhibition, MEK inhibition, and PD-1/PD-L1 inhibition with the two-drug combination regimen or monotherapy alone in patients with metastatic melanoma, thereby providing a reliable assessment of the role of triple therapy in advanced melanoma disease. Meta-analysis has been recognized as an effective method to assess the totality of the available data, thus avoiding selective emphasis and providing answers to controversial or unresolved clinical questions.

The random-effects model of our meta-analysis demonstrated that the triple combination therapy resulted in an evident improvement in progression-free survival compared with control group. Also, overall survival rates were significantly higher in patients treated with triple therapy compared with those receiving control therapy. However, there was no difference between the triple combination therapy and control treatment regimen with regard to the overall response rate, and the results of ORR showed high heterogeneity. When the ORR was split into complete response and partial response, there were still no significant differences between these two treatments and the high heterogeneity of ORR was mainly caused by the partial response. Therefore, despite better PFS and OS outcomes in those patients receiving triple combination therapy, one thing needs to be noted that the overall response rate also served as an important therapeutic outcome which might be useful for better symptom control in certain clinical situations (28). In terms of safety, triple combination therapy did not increase the overall incidence of any AEs or grade ≥3 AEs, but the occurrence rates of hypothyroidism, arthralgia, myalgia, ALT increased, AST increased, asthenia, and pyrexia were significantly higher than in the control group.

BRAF and MEK inhibition generally resulted in higher ORR for patients, whereas PD-1/PD-L1 inhibition typically resulted in more durable responses. Because these treatments have distinct mechanisms of action in melanoma, triple therapy combining PD-1/PD-L1 inhibitors with BRAF and MEK inhibitors were proposed and gradually used in preclinical models and clinical trials (29, 30). However, meta-analysis from studies that directly compare the triple combination therapy with two-drug combination or monotherapy is lacking. Past studies mainly focused on examining the efficacy and adverse events of standard-of-care therapy such as BRAF inhibitor plus a MEK inhibitor (31, 32), anti-PD-1/PD-L1 monotherapy (33), and nivolumab (anti-PD-1) plus ipilimumab (anti-CTLA-4) combination (34). Although these treatments have improved patient survival, the problems of drug resistance to targeted therapies and low response rates to immunotherapies still need to be addressed. To our knowledge, this study is the first meta-analysis that systematically summarizes and analyzes the safety and efficacy of triple combination therapy as compared to two-drug combinations or monotherapy for advanced melanoma.

Triple combination therapy was associated with promising anti-tumor activity. When targeted therapy begins, a series of changes in the immune microenvironment will occur, which is conducive to subsequent immunotherapy. Previous mouse model of syngeneic *BRAF* ^V600E^ driven melanoma showed that combination of BRAF and MEK inhibitor (dabrafenib and trametinib) increased T cell infiltration into tumors, up-regulated PD-L1 expression and improved *in vivo* cytotoxicity. Triple combination of dabrafenib, trametinib with anti-PD-1 therapy offered superior anti-tumor effect in *BRAF* ^V600E^ murine melanoma (30). In a phase Ib study (35), patients treated with BRAF and MEK inhibitor (vemurafenib and cobimetinib) also induced changes in the tumor microenvironment, including the increased proportion of CD8^+^ T cells and increased proliferation of CD4^+^ T-helper cells, these favorable changes may enhance response to anti-PD-1/PD-L1 immunotherapy. Immunohistochemistry analysis and RNA sequencing data showed increased MHC class I expression and immune infiltration in triple therapy (17, 36). Therefore, these molecular mechanisms of triple combination therapy provided a feasible treatment approach for advanced melanoma.

The primary endpoint of investigator-assessed PFS in the IMSpire 150 study exhibited a significant improvement of 4.5 months difference (15.1 *vs*. 10.6 months) in the triple combination therapy, which led to FDA approval of this triple therapy (atezolizumab, vemurafenib and cobimetinib) for treatment of *BRAF* ^V600^ mutation-positive melanoma. In terms of treatment therapies, IMSpire 150 study designed a run-in period of targeted therapy prior to the initiation of PD-L1 immunotherapy, which differed from the Keynote-022 and COMBI-I study that used three-drug combinations at the same time. The smart design resulted in better drug tolerance among patients in the triple therapy group of IMSpire 150 study. Therefore, we recommended introducing a run-in period of BRAF and MEK inhibitors before PD-1/PD-L1 immunotherapy to maximize the benefits of triple therapy. In addition, more clinical trials are required to find out the optimal triple combination regimen and administration time.

This study has several limitations that deserve to be mentioned. The first drawback is that the patients in the included studies were treated with different triple combinations and patients in the control arm also received different treatment regimens. Second, because the triple combination therapy is emerging and has been applied in clinical practice only in recent years, some studies such as Immu-Target (NCT02902042) (37) and NeoTrio (NCT02858921) (38) are still at the preliminary stage, therefore, the numbers of included studies were relatively small. The limited number of studies and patients for analysis could probably lead to decreased accuracy and reliability of our comparison results. To overcome this problem, we conducted sensitivity analysis to test the robustness of the results. Third, high heterogeneity existed in overall response rates, partial response and many adverse events. Our meta-analysis included phase 1, phase 2, and phase 3 clinical trials could introduce heterogeneity among the results. To overcome this issue, random-effects model was adopted in all analyses because unexplained heterogeneity was taken into account in this model.

## Conclusion

In conclusion, triple combination therapy of PD-1/PD-L1, BRAF, and MEK inhibition had significant survival benefit over two-drug combination or monotherapy and should be a new preferred therapy for patients with stage III-IV advanced and metastatic melanoma, as confirmed by the present meta-analysis. Attention should be paid to some adverse events and physicians need to balance against the increased toxicity when using triple combination therapy. Utilizing the triple combination drugs sensitized the patients’ immune system to improve the effectiveness of immunotherapy and inhibited BRAF plus MEK to control tumor growth. From our results, we conclude that triple combination therapy has great benefits for patients harboring the *BRAF* ^V600^ mutation-positive advanced melanoma, and designing a run-in period of BRAF and MEK targeted therapy prior to the initiation of PD-1/PD-L1 immunotherapy could maximize the benefits of triple therapy. Despite the promising results of triple combination therapy, longer follow-up is required to see the possible benefit from the triple therapy. Besides that, more clinical trials are required to determine which patients benefit the most from the triple therapy, and to find out the optimal sequence and dose of triple drug administration. We hope this current meta-analysis could provide a reference point for physicians in clinical treatment when considering the optimum combination regimen for advanced melanoma patients.

## Data Availability Statement

The original contributions presented in the study are included in the article/**Supplementary Material**. Further inquiries can be directed to the corresponding author.

## Author Contributions

Concept and design: YL, XC. Acquisition, analysis, or interpretation of data: All authors. Drafting of the manuscript: YL, XC. Critical revision of the manuscript for important intellectual content: YL, XC. Statistical analysis: YL, GW, XZ. Administrative, technical, or material support: YL, XZ, GW. Supervision: GW, XC. All authors contributed to the article and approved the submitted version.

## Conflict of Interest

The authors declare that the research was conducted in the absence of any commercial or financial relationships that could be construed as a potential conflict of interest.

## Supplementary Material

Supplementary Figure 1 Sensitivity analysis of progression free survival (PFS).

Supplementary Figure 2 Sensitivity analysis of overall survival (OS).

Supplementary Figure 3 Sensitivity analysis of overall response rate (ORR)

Supplementary Figure 4 Sensitivity analysis of complete response (CR).

Supplementary Figure 5 Sensitivity analysis of partial response (PR).

Supplementary Figure 6 Sensitivity analysis of adverse events for all grades.

Supplementary Figure 7 Sensitivity analysis of adverse events for grade≥ 3.

Supplementary Table 1 Search Strategies.
