# Supplementary figures and images for: Triple Combination Therapy With PD-1/PD-L1, BRAF, and MEK Inhibitor for Stage III–IV Melanoma: A Systematic Review and Meta-Analysis
Source: Front Oncol. 2021 Jun 14;11:693655. doi: 10.3389/fonc.2021.693655 (PMC8236832; doi:10.3389/fonc.2021.693655)

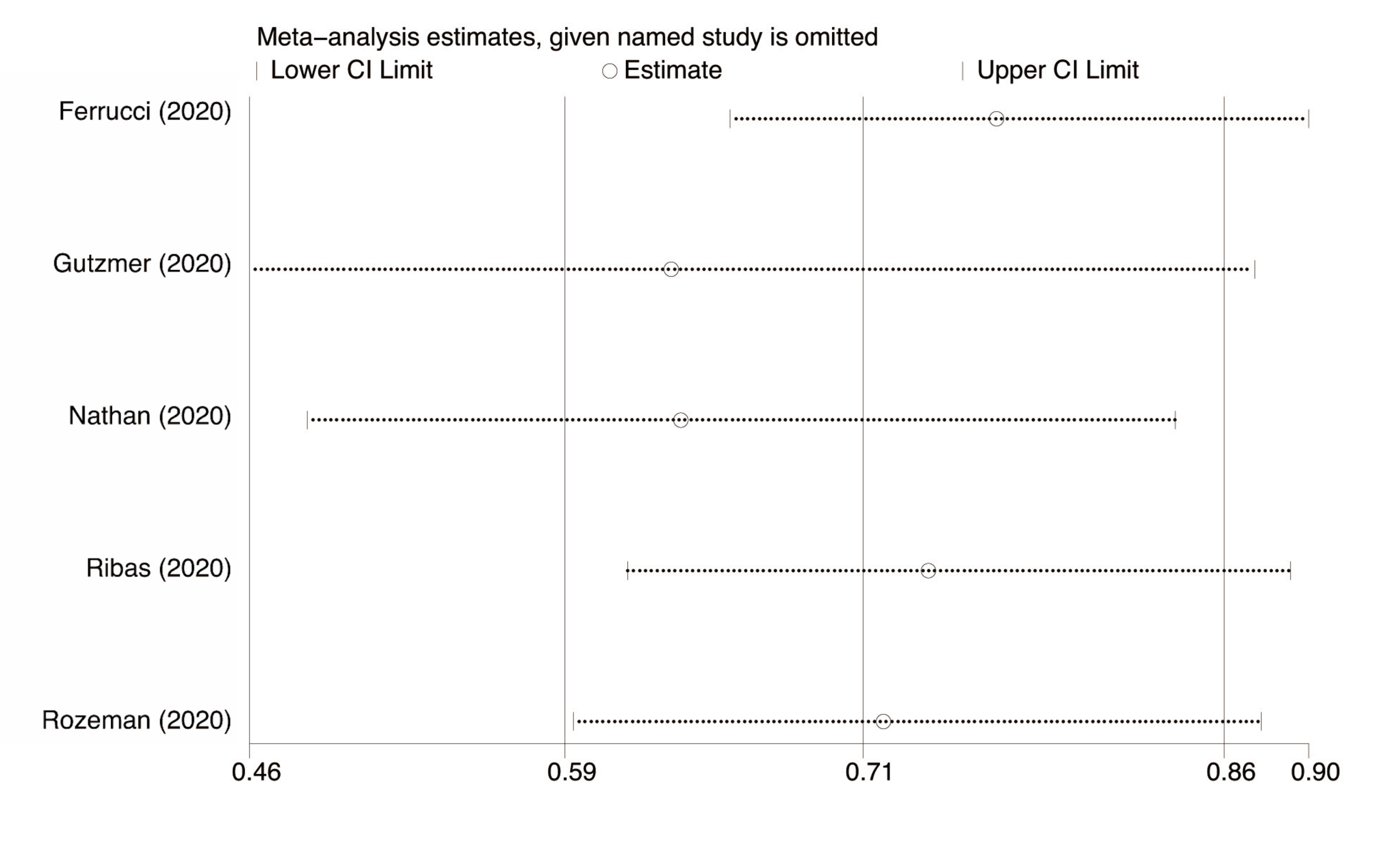

Supplement: Supplementary Figure 1 — Sensitivity analysis of progression free survival (PFS). [file Image_1.tif]

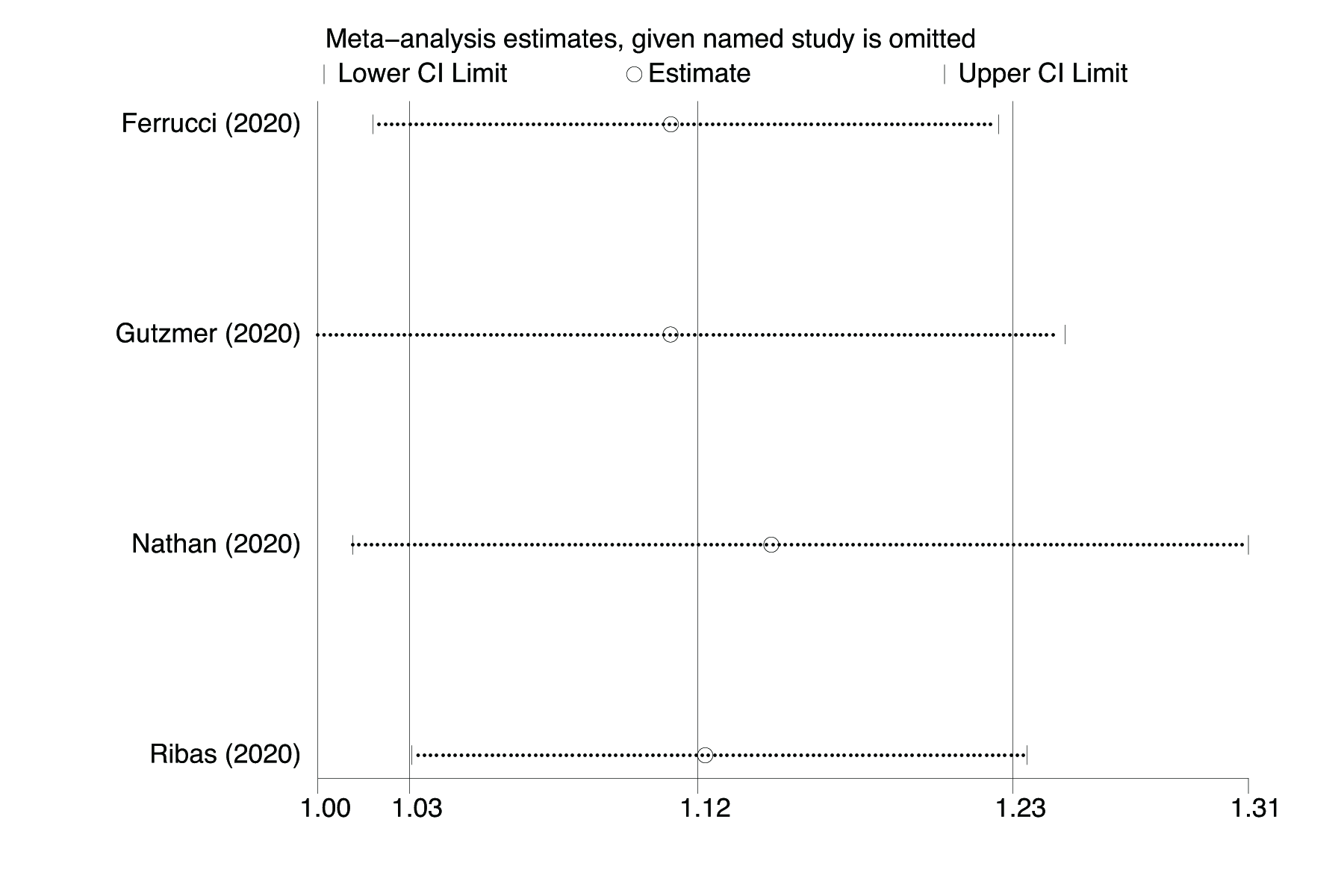

Supplement: Supplementary Figure 2 — Sensitivity analysis of overall survival (OS). [file Image_2.tif]

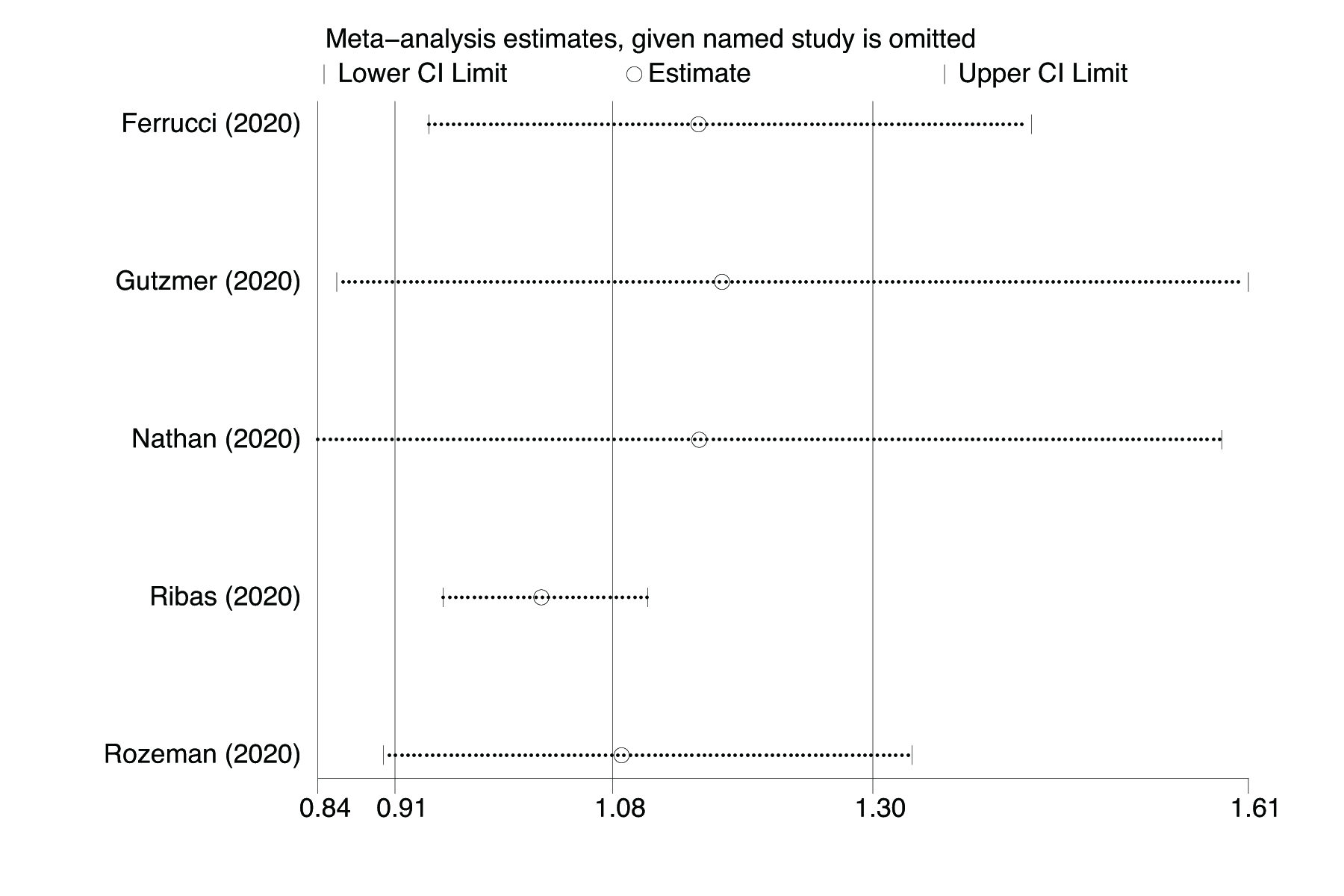

Supplement: Supplementary Figure 3 — Sensitivity analysis of overall response rate (ORR) [file Image_3.tif]

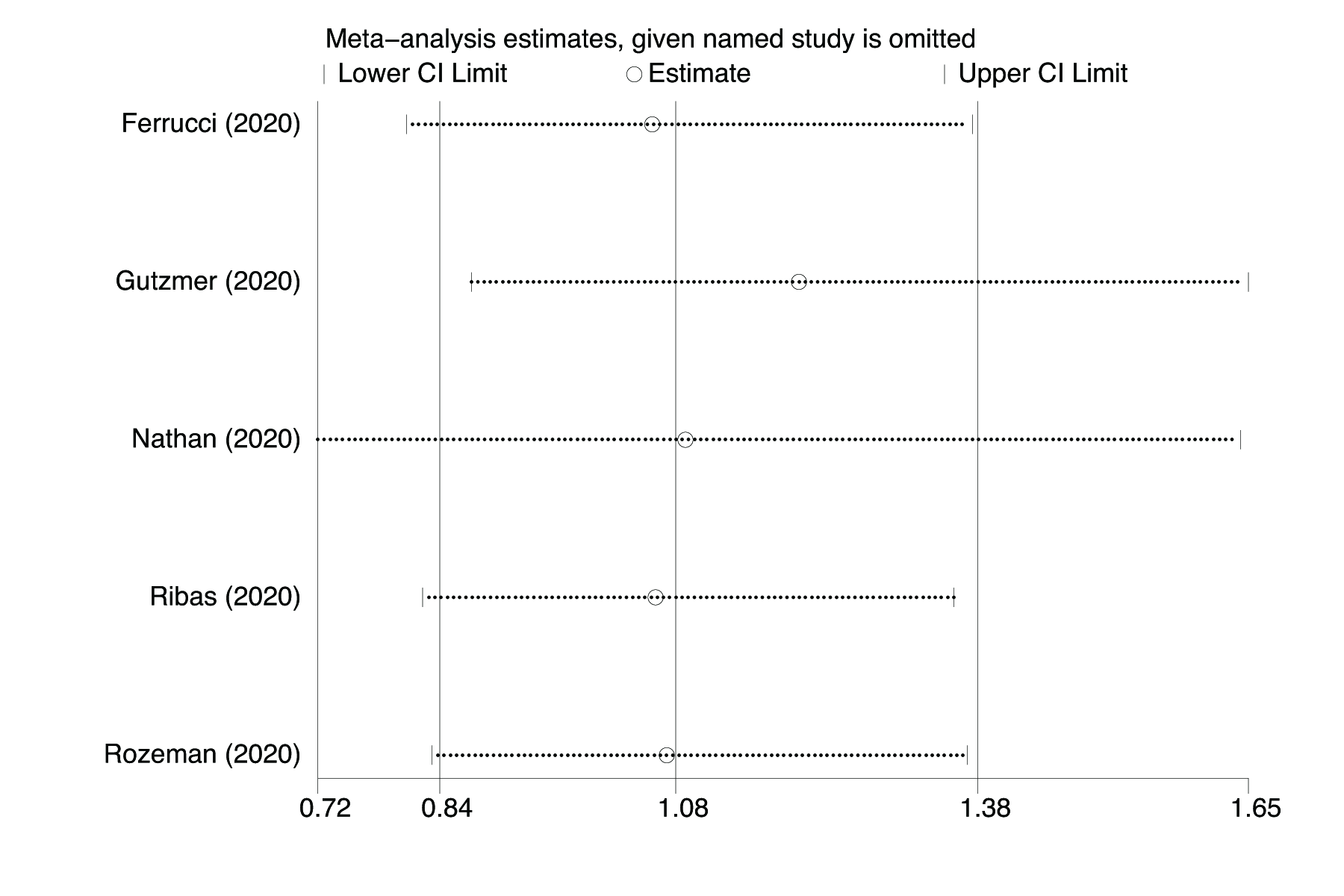

Supplement: Supplementary Figure 4 — Sensitivity analysis of complete response (CR). [file Image_4.tif]

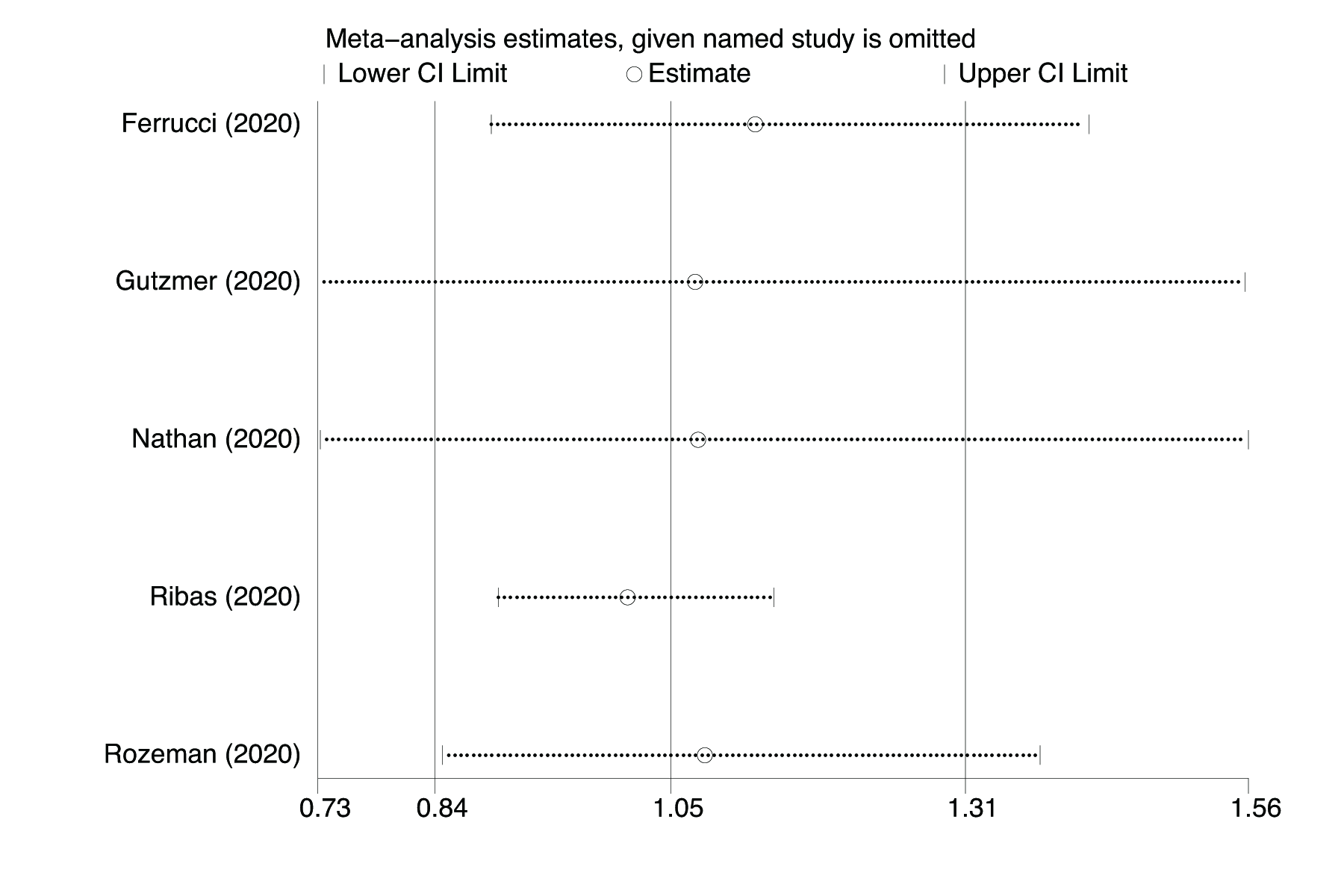

Supplement: Supplementary Figure 5 — Sensitivity analysis of partial response (PR). [file Image_5.tif]

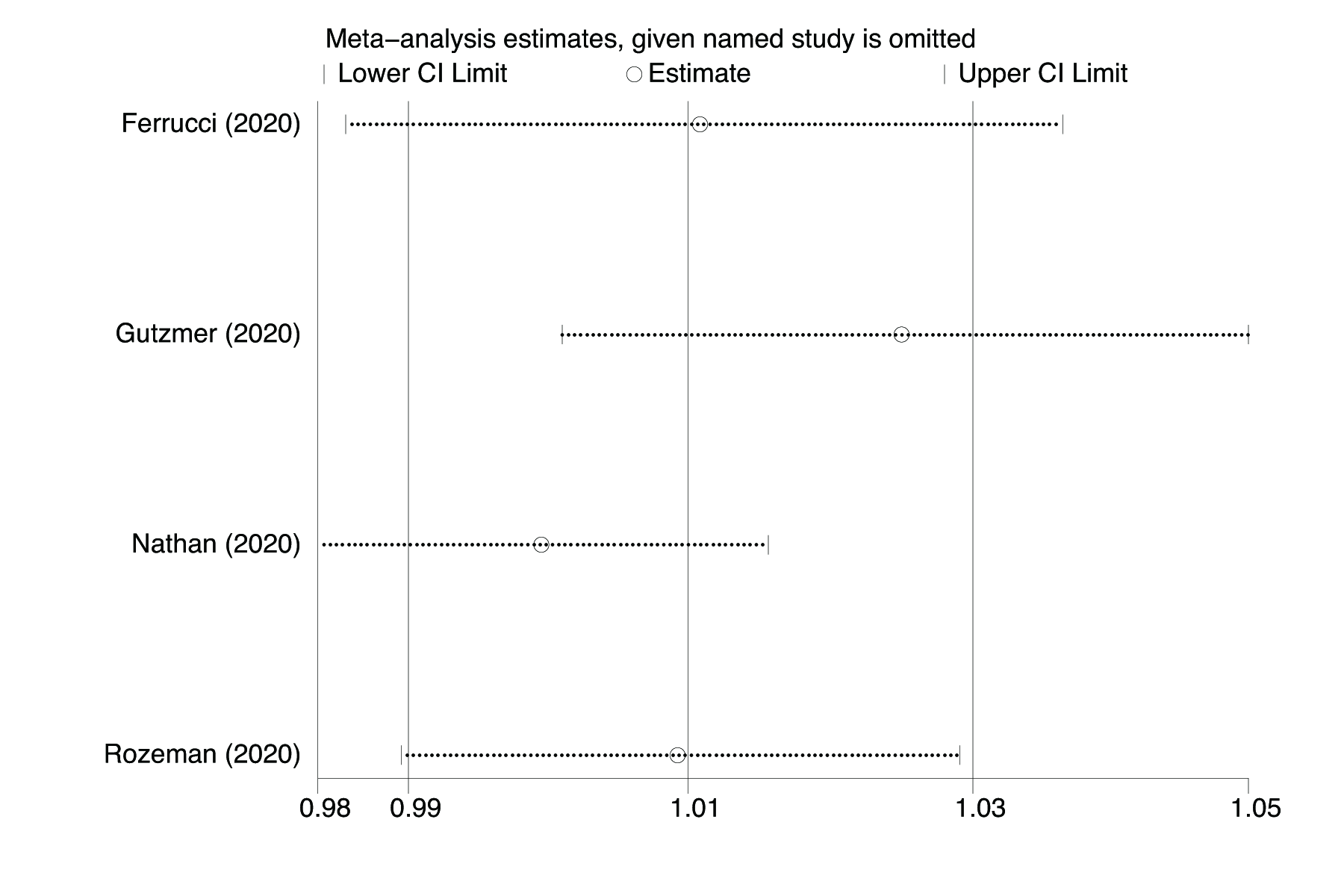

Supplement: Supplementary Figure 6 — Sensitivity analysis of adverse events for all grades. [file Image_6.tif]

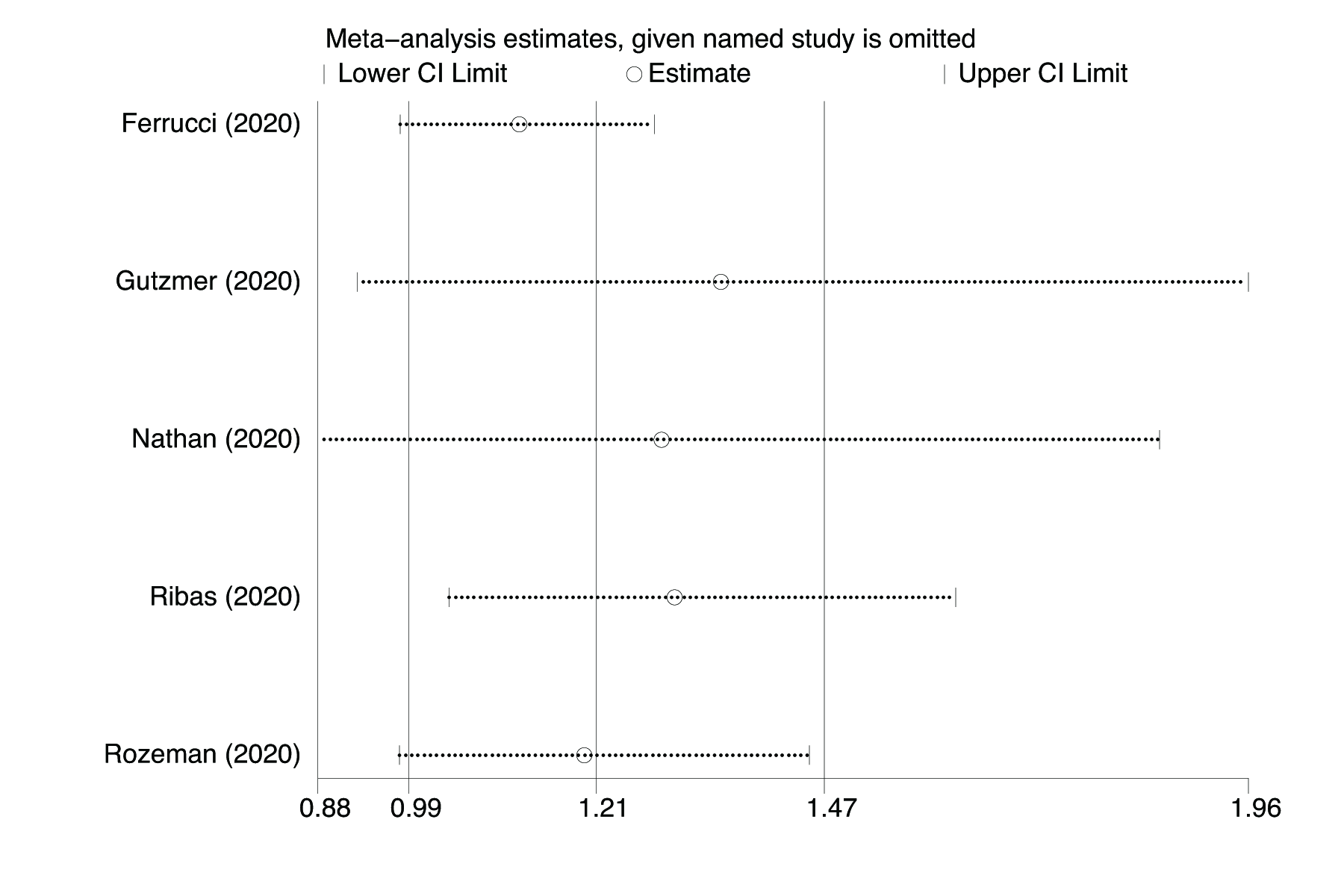

Supplement: Supplementary Figure 7 — Sensitivity analysis of adverse events for grade≥ 3. [file Image_7.tif]
