# Supplementary material for: Triple Combination Therapy With PD-1/PD-L1, BRAF, and MEK Inhibitor for Stage III–IV Melanoma: A Systematic Review and Meta-Analysis
Source: Front Oncol. 2021 Jun 14;11:693655. doi: 10.3389/fonc.2021.693655 (PMC8236832; doi:10.3389/fonc.2021.693655)
Supplement: Supplementary Table 1 — Search Strategies. [file Table_1.doc]

Search strategies

| **Pubmed 87** | #1 "melanoma" [MeSH]  #2 "skin neoplasms" [MeSH]  #3 "Carcinoma, Basal Cell" [MeSH]  #4 melanoma*[Title/Abstract] OR basalioma*[Title/Abstract]  #5 (basal cell [Title/Abstract] OR skin[Title/Abstract]) AND (cancer*[Title/Abstract] OR cacinoma*[Title/Abstract] OR mass*[Title/Abstract] OR tumour*[Title/Abstract] OR tumor*[Title/Abstract] OR neoplas*[Title/Abstract] OR adenoma*[Title/Abstract] OR epithelioma*[Title/Abstract] OR maligan*[Title/Abstract] OR nodule*[Title/Abstract])  #6 pigmented [Title/Abstract] AND (lesion*[Title/Abstract] OR mole*[Title/Abstract] OR nevus[Title/Abstract] OR nevi[Title/Abstract] OR naevus[Title/Abstract] OR naevi[Title/Abstract] OR skin[Title/Abstract])  #7 #1 OR #2 OR #3 OR #4 OR #5 OR #6  #8 "Programmed Cell Death 1 Receptor"[MeSH]  #9 programmed death 1[Title/Abstract] OR programmed death-ligand 1[Title/Abstract] OR PD-1[Title/Abstract] OR PD-L1[Title/Abstract]  #10 pembrolizumab[Title/Abstract] OR lambrolizumab[Title/Abstract] OR MK-3475[Title/Abstract] OR Keytruda[Title/Abstract]  #11 pidilizumab[Title/Abstract] OR CT-011[Title/Abstract]  #12 "Nivolumab"[MeSH] OR opdivo*[Title/Abstract] OR nivo*[Title/Abstract] OR BMS‐936558[Title/Abstract] OR MDX‐1106[Title/Abstract] OR ONO‐4538[Title/Abstract] OR BMS936558[Title/Abstract] OR MDX1106[Title/Abstract]  #13 atezolizumab[Title/Abstract] OR MPDL3280*[Title/Abstract] OR MPDL-3280A[Title/Abstract] OR Tecentriq[Title/Abstract] OR RG7446[Title/Abstract] OR RG-7446[Title/Abstract]  #14 durvalumab[Title/Abstract] OR Imfinzi[Title/Abstract] OR MEDI4736[Title/Abstract] OR MEDI-4736[Title/Abstract]  #15 avelumab[Title/Abstract] OR Bavencio[Title/Abstract] OR MSB0010718C[Title/Abstract]  #16 Spartalizumab[Title/Abstract] OR PDR001[Title/Abstract]  #17 #8 OR #9 OR #10 OR #11 OR #12 OR #13 OR #14 OR #15 OR #16  #18 "Proto-Oncogene Proteins B-raf"[MeSH] OR BRAF [Title/Abstract]  #19 "Vemurafenib" [MeSH]  #20 PLX 4032[Title/Abstract] OR RG7204[Title/Abstract] OR RG-7204[Title/Abstract] OR Zelboraf[Title/Abstract]  #21 dabrafenib [Title/Abstract] OR GSK-2118436[Title/Abstract] OR Tafinlar [Title/Abstract]  #22 encorafenib [Title/Abstract] OR LGX818[Title/Abstract] OR Braftovi [Title/Abstract]  #23 #18 OR #19 OR #20 OR #21 OR #22  #24 "Mitogen-Activated Protein Kinases "[MeSH] OR "Mitogen-Activated Protein Kinase Kinases"[MeSH] OR MEK[Title/Abstract] OR MAPK[Title/Abstract]  #25 trametinib [Title/Abstract] OR JTP-74057[Title/Abstract] OR GSK1120212[Title/Abstract] OR GSK-1120212[Title/Abstract] OR Mekinist [Title/Abstract]  #26 cobimetinib [Title/Abstract] OR Cotellic [Title/Abstract] OR GDC-0973[Title/Abstract]  #27 binimetinib [Title/Abstract] OR Mektovi [Title/Abstract] OR MEK162[Title/Abstract]  #28 selumetinib [Title/Abstract] OR AZD6244[Title/Abstract] OR AZD-6244[Title/Abstract] OR ARRY142886[Title/Abstract] OR ARRY-142886[Title/Abstract]  #29 #24 OR #25 OR #26 OR #27 OR #28  #30 (randomized controlled trial [Publication Type] OR controlled clinical trial [Publication Type] OR randomized [Title/Abstract] OR placebo [Title/Abstract] OR clinical trials as topic [MeSH: noexp] OR randomly [Title/Abstract] OR trial [Title]) NOT (animals [MeSH Terms] NOT humans [MeSH Terms])  #31 #7 AND #17 AND #23 AND #29 AND #30 |
| --- | --- |
| **Cochrane 115** | #1 MeSH descriptor: [Melanoma] explode all trees  #2 MeSH descriptor: [Skin Neoplasms] explode all trees  #3 MeSH descriptor: [Carcinoma, Basal Cell] explode all trees  #4 (melanoma*):ti,ab,kw OR (basalioma*):ti,ab,kw  #5 ((basal cell OR skin) AND (cancer* OR cacinoma* OR mass* OR tumour* OR tumor* OR neoplas* OR adenoma* OR epithelioma* OR maligan* OR nodule*)):ti,ab,kw  #6 (pigmented AND (lesion* OR mole* OR nevus OR nevi OR naevus OR naevi OR skin)):ti,ab,kw  #7 #1 OR #2 OR #3 OR #4 OR #5 OR #6  #8 MeSH descriptor: [Programmed Cell Death 1 Receptor] explode all trees  #9 ((programmed death 1) OR (programmed death-ligand 1) OR (PD-1) OR (PD-L1)):ti,ab,kw  #10 (pembrolizumab OR lambrolizumab OR MK-3475 OR Keytruda):ti,ab,kw  #11 (pidilizumab OR (CT-011)):ti,ab,kw  #12 MeSH descriptor: [Nivolumab] explode all trees  #13 (opdivo* OR nivo* OR BMS‐936558 OR MDX‐1106 OR ONO‐4538 OR BMS936558 OR MDX1106):ti,ab,kw  #14 ((atezolizumab) OR (MPDL3280*) OR (MPDL-3280A) OR (Tecentriq) OR (RG7446) OR (RG-7446)):ti,ab,kw  #15 ((durvalumab) OR (Imfinzi) OR (MEDI4736) OR (MEDI-4736)):ti,ab,kw  #16 ((avelumab) OR (Bavencio) OR (MSB0010718C)):ti,ab,kw  #17 ((Spartalizumab) OR (PDR001)):ti,ab,kw  #18 #8 OR #9 OR #10 OR #11 OR #12 OR #13 OR #14 OR #15 OR #16 OR #17  #19 MeSH descriptor: [Proto-Oncogene Proteins B-raf] explode all trees  #20 MeSH descriptor: [Vemurafenib] explode all trees  #21 ((BRAF) OR (PLX 4032) OR (RG7204) OR (RG-7204) OR (Zelboraf)):ti,ab,kw  #22 ((dabrafenib) OR (GSK-2118436) OR (Tafinlar)):ti,ab,kw  #23 ((encorafenib) OR (LGX818) OR (Braftovi)):ti,ab,kw  #24 #19 OR #20 OR #21 OR #22 OR #23  #25 MeSH descriptor: [Mitogen-Activated Protein Kinases] explode all trees  #26 MeSH descriptor: [Mitogen-Activated Protein Kinase Kinases] explode all trees  #27 (MEK OR MAPK):ti,ab,kw  #28 ((trametinib) OR (JTP-74057) OR (GSK1120212) OR (GSK-1120212) OR (Mekinist)):ti,ab,kw  #29 ((cobimetinib) OR (Cotellic) OR (GDC-0973)):ti,ab,kw  #30 ((binimetinib) OR (Mektovi) OR (MEK162)):ti,ab,kw  #31 ((selumetinib) OR (AZD6244) OR (AZD-6244) OR (ARRY142886) OR (ARRY-142886)):ti,ab,kw  #32 #25 OR #26 OR #27 OR #28 OR #29 OR #30 OR #31  #33 #7 AND #18 AND #24 AND #32 |
| **Embase 240** | #1 'melanoma'/exp  #2 'skin tumor'/exp  #3 'basal cell carcinoma'/exp  #4 melanoma*:ab,ti OR basalioma*:ab,ti  #5 ('basal cell':ab,ti OR skin:ab,ti) AND (cancer*:ab,ti OR cacinoma*:ab,ti OR mass*:ab,ti OR tumour*:ab,ti OR tumor*:ab,ti OR neoplas*:ab,ti OR adenoma*:ab,ti OR epithelioma*:ab,ti OR maligan*:ab,ti OR nodule*:ab,ti)  #6 pigmented:ab,ti AND (lesion*:ab,ti OR mole*:ab,ti OR nevus:ab,ti OR nevi:ab,ti OR naevus:ab,ti OR naevi:ab,ti OR skin:ab,ti)  #7 #1 OR #2 OR #3 OR #4 OR #5 OR #6  #8 'programmed death 1 receptor'/exp  #9 'programmed death 1 ligand 1'/exp  #10 'programmed death 1':ab,ti OR 'programmed death-ligand 1':ab,ti OR 'pd 1':ab,ti OR 'pd l1':ab,ti  #11 pembrolizumab:ab,ti OR lambrolizumab:ab,ti OR 'mk 3475':ab,ti OR keytruda:ab,ti  #12 pidilizumab:ab,ti OR 'ct 011':ab,ti  #13 'nivolumab'/exp  #14 opdivo*:ab,ti OR nivo*:ab,ti OR bms‐936558:ab,ti OR mdx‐1106:ab,ti OR ono‐4538:ab,ti OR bms936558:ab,ti OR mdx1106:ab,ti  #15 atezolizumab:ab,ti OR mpdl3280*:ab,ti OR 'mpdl 3280a':ab,ti OR tecentriq:ab,ti OR rg7446:ab,ti OR 'rg 7446':ab,ti  #16 durvalumab:ab,ti OR imfinzi:ab,ti OR medi4736:ab,ti OR 'medi 4736':ab,ti  #17 avelumab:ab,ti OR bavencio:ab,ti OR msb0010718c:ab,ti  #18 spartalizumab:ab,ti OR pdr001:ab,ti  #19 #8 OR #9 OR #10 OR #11 OR #12 OR #13 OR #14 OR #15 OR #16 OR #17 OR #18  #20 'b raf kinase'/exp  #21 'vemurafenib'/exp  #22 braf:ab,ti OR 'plx 4032':ab,ti OR rg7204:ab,ti OR 'rg 7204':ab,ti OR zelboraf:ab,ti  #23 dabrafenib:ab,ti OR 'gsk 2118436':ab,ti OR tafinlar:ab,ti  #24 encorafenib:ab,ti OR lgx818:ab,ti OR braftovi:ab,ti  #25 #20 OR #21 OR #22 OR #23 OR #24  #26 'mitogen activated protein kinase'/exp  #27 'mitogen activated protein kinase kinase'/exp  #28 mek:ab,ti OR mapk:ab,ti  #29 trametinib:ab,ti OR 'jtp 74057':ab,ti OR gsk1120212:ab,ti OR 'gsk 1120212':ab,ti OR mekinist:ab,ti  #30 cobimetinib:ab,ti OR cotellic:ab,ti OR 'gdc 0973':ab,ti  #31 binimetinib:ab,ti OR mektovi:ab,ti OR mek162:ab,ti  #32 selumetinib:ab,ti OR azd6244:ab,ti OR 'azd 6244':ab,ti OR arry142886:ab,ti OR 'arry 142886':ab,ti  #33 #26 OR #27 OR #28 OR #29 OR #30 OR #31 OR #32  #34 'crossover procedure':de OR 'double-blind procedure':de OR 'randomized controlled trial':de OR 'single-blind procedure':de OR random*:de,ab,ti OR factorial*:de,ab,ti OR crossover*:de,ab,ti OR ((cross NEXT/1 over*):de,ab,ti) OR placebo*:de,ab,ti OR ((doubl* NEAR/1 blind*):de,ab,ti) OR ((singl* NEAR/1 blind*):de,ab,ti) OR assign*:de,ab,ti OR allocat*:de,ab,ti OR volunteer*:de,ab,ti  #35 #7 AND #19 AND #25 AND #33 AND #34 |
